# The Responders’ Gender Stereotypes Modulate the Strategic Decision-Making of Proposers Playing the Ultimatum Game

**DOI:** 10.3389/fpsyg.2016.00012

**Published:** 2016-01-25

**Authors:** Eve F. Fabre, Mickael Causse, Francesca Pesciarelli, Cristina Cacciari

**Affiliations:** ^1^Department of Biomedical, Metabolic and Neural Sciences, University of Modena and Reggio EmiliaModena, Italy; ^2^Institut Supérieur de l’Aéronautique et de l’EspaceToulouse, France

**Keywords:** ultimatum game, gender stereotypes, proposer, strategic decision-making

## Abstract

Despite the wealth of studies investigating factors affecting decisions, not much is known about the impact of stereotypical beliefs on strategic economic decision-making. In the present study, we used the ultimatum game paradigm to investigate how participants playing as proposer modulate their strategic economic behavior, according to their game counterparts’ stereotypical identity (i.e., responders). The latter were introduced to the participants using occupational role nouns stereotypically marked with gender paired with feminine or masculine proper names (e.g., linguist-Anna; economist-David; economist-Cristina; linguist-Leonardo). When playing with male-stereotyped responders, proposers quickly applied the equity rule, behaving fairly, while they adopted a strategic behavior with responders characterized by female stereotypes. They were also longer to make their offers to female than to male responders but both kinds of responders received comparable offers, suggesting a greater cognitive effort to treat females as equally as males. The present study explicitly demonstrates that gender stereotypical information affect strategic economic decision-making and highlights a possible evolution of gender discrimination into a more insidious discrimination toward individuals with female characteristics.

## Introduction

### The Proposer Playing the Ultimatum Game: Between Social Preference and Strategy

One topic of major interest in economic decision-making studies is the strategic behavior adopted by individuals faced with economic decisions (Camerer, 2003). The interest in how social and emotional information affects economic decision-making has steadily grown over past decades (for overviews see Frith and Singer, 2008; Rilling and Sanfey, 2011). The ultimatum game has provided, for now many years, a fruitful paradigm for assessing the social aspects of economic decision-making (Güth et al., 1982). In the standard version of this two-player game, a proposer offers to split a fixed amount of money (e.g., 10€) with a responder. Both receive their shares only if the responder accepts the offer. Game theory predicts that in order to maximize their outcome, proposers should behave in a rational and self-interested way, offering the smallest share possible to the responder (e.g., 1€ out of 10€; Neumann and Morgenstern, 1947). But psychological research on judgment and decision-making has produced a wealth of evidence that, in practice, this theory does not provide a satisfactory description of human behavior (e.g., Güth et al., 1982; Camerer, 2003). Indeed, on average, proposers offer about 40% of the total amount of money (e.g., 4€ out of 10€) to the responders (for a meta-analysis see Oosterbeek et al., 2004). Such a behavior has been under scrutiny for now decades, and various theories have been proposed to explain the proposers’ fair behavior.

Social norms are defined as “the customary rules that govern behavior in groups and societies.” Honesty, loyalty, reciprocity, or promise keeping, to name a few, do guarantee smooth interactions between individuals of a given social group or society (Bicchieri, 2006). This common value system plays a crucial role in individual choices because, by shaping individual needs and preferences, these norms serve as criteria for selecting among alternatives. People choose what they prefer, and what they prefer conforms most of the time to social expectations (Bicchieri, 2006). According to *social preference* models (Fehr and Schmidt, 1999; Falk and Fischbacher, 2006), proposers, who embrace social norms, make almost fair offers because they have altruistic concerns toward the responders and care about the distribution of payoffs among players. On the other hand, according to other authors, the apparent fairness preference of the proposers may in turn reflect strategic concerns (for an overview see Nelissen et al., 2011). Indeed, the proposers are aware that responders are likely to have social expectations and thus may reject unfair offers (Güth et al., 1982; Camerer, 1999, 2003; Sanfey, 2009). Consequently, because they want to maximize their gain, the proposers have to accurately determine the smallest amount of money the responders may accept (i.e., the minimum acceptable; Blount, 1995). Then, proposers may feign altruism, offering almost fair shares to the responders (Kagel et al., 1996; Nelissen et al., 2011), aiming at lowering the occurrence of emotionally painful rejections (Güroǧlu et al., 2009). The results of various studies attest that it is more likely that proposers’ fair behavior reflects both altruistic and strategic concerns (Blount, 1995), with a certain inter-variability among individuals in the altruistic/strategic balance (Morishima et al., 2013).

While rarely reported in the ultimatum game studies, the time needed by the participants to make a decision (i.e., response times) may be a good indicator of both the complexity of the decision and the cognitive processes involved in the decision-making. Recent studies (e.g., Polezzi et al., 2008; Fabre et al., 2015) found that responders playing the ultimatum game were faster answering easily classifiable offers, both fair (i.e., 5€ out of 10€) and unfair (i.e., 1€ out of 10€), than hardly classifiable mid-value offers (i.e., 3€ of 10€; Sanfey et al., 2003). The decision on both unfair and fair offers appears to rely on fast heuristic-based judgments (i.e., refusing unfair offers and accepting fair offers), while deciding on mid-value offers may be more complex (Sanfey et al., 2003) and therefore requires a more time consuming and cognitively costly deliberative reasoning (Civai, 2013; Fabre et al., 2015). It is plausible that the proposers’ decision-making works in a similar way. Indeed, when proposers have social preferences, their decision-making may be relatively fast, since they would follow social norms and apply the equity rule (i.e., offering a fair share). In contrast, when proposers engage in a strategic decision-making, they have to accurately evaluate all the information available to maximize their gain, which may be cognitively costly and time consuming. To this extent, we assume that studying the proposers’ response times may provide critical information concerning the decision process and the balance between altruistic and strategic concerns.

### The Impact of Social Information on the Proposers’ Strategic Concerns

Several studies investigated how proposers modulate their behavior depending on the responders’ social characteristics (e.g., Solnick and Schweitzer, 1999). These studies allowed us to evaluate the proposers’ internal representations of the world. In other words, how much is a specific responder “worth” to a proposer? Eckel and Ball (1996) investigated the effect of social status information on the proposers’ behavior. In their study, participants were attributed a star or not depending on their performance to a trivia quiz before playing the ultimatum game as either proposer or responder. The priming task (i.e., trivia quiz) enabled to allocate artificially a high (i.e., star) or a low status (i.e., no star) to the participants. Both high and low status proposers offered higher shares to high status responders than to low social status responders, confirming the impact of social status on economic decision-making (for an overview see Heffetz and Frank, 2008). According to *status characteristic* theory (Wagner and Berger, 1993), a status characteristic (i.e., gender, age, race, physical attractiveness, intelligence or occupation) affects people’s expectations of reward, and high status individuals expect to receive higher reward than low status individuals. To this extent, proposers may adapt their behavior, offering higher shares to high status responders compared with the low status responders in order to limit the risk of suffering a rejection (Güroǧlu et al., 2009). Proposers were also found to be influenced by the responders’ attractiveness offering higher shares to more attractive responders (Solnick and Schweitzer, 1999; Zaatari et al., 2009). Finally, some studies investigated the impact of the responders’ gender on the proposers’ decision. Overall, these studies demonstrated that proposers offer more to male responders than to female responders (e.g., Solnick and Schweitzer, 1999; Eckel and Grossman, 2001; Solnick, 2001). Saad and Gill (2001) found that male proposers were on average more generous with female than male responders, while the responders’ gender did not affect the behavior of female proposers, who offered equally fair shares to responders of both sexes. A greater variability in the behavior of male proposers was also found with altruistic male proposers or aggressive male proposers compared to female proposers, who showed less variability in their behavior (Castillo and Cross, 2008).

Because both gender and attractiveness are considered as status characteristics, the observation of the increased shares proposed to attractive responders (Solnick and Schweitzer, 1999; Zaatari et al., 2009) and to male responders in most gender studies (e.g., Solnick and Schweitzer, 1999; Eckel and Grossman, 2001; Solnick, 2001) may be interpreted in terms of social status differences.

### Why Investigate the Impact of Gender Stereotypes on Economic Decision-Making?

Conflict theories postulate that because men have greater social status and power (Reskin, 1988), they allocate occupations that open access to resources (e.g., money, stocks, contacts, information) predominantly to men, thus favoring themselves over women, which creates an occupational segregation (Pratto et al., 1997). For this reason, stereotypically male occupations (e.g., engineer, electrician) are associated with higher social status and power compared with those stereotypically female (e.g., teacher, beautician; Eagly, 1987; Ridgeway, 2001). Nowadays, more and more women can enter the professions with a highly marked male stereotype (e.g., lawyer, banker, doctor), and more men access to occupations with a highly marked female stereotype (e.g., nurse, “mid-wife,” teacher; Eagly, 1987; Eagly and Karau, 2002; Phelan et al., 2008). Given the frequent interactions of men and women, it is critical to understand how gender stereotypical beliefs – i.e., a form of social knowledge linked to actions, attitudes, rules and other forms of knowledge attributed to individuals based on their biological gender (Greenwald and Farnham, 2000; Wheeler and Petty, 2001; Quadflieg and Macrae, 2011) – modulate economic decision-making.

A recent study investigated how proposers’ gender stereotyped descriptions (i.e., occupations marked with either a male or a female stereotype) influenced the responders’ decision-making (Fabre et al., 2015). When playing with female-stereotyped proposers (e.g., linguist), responders were longer to make their decision, reflecting a more deliberative reasoning (Sanfey and Chang, 2008) associated with an increase in acceptation rates. In contrast, participants were found to answer more quickly and to reject more frequently male-stereotyped proposers’ offers (e.g., economist) than those of female-stereotyped proposers. That study demonstrated that gender stereotype information of the proposer modulates the economic decision-making in the ultimatum game and the cognitive processes underpinning the decision-making. Therefore, we may reasonably expect gender stereotypical information of responders to modulate the behavior of proposers playing the ultimatum game. To our knowledge, this impact of the responders’ stereotypical identity on the proposers’ decision-making has never been investigated.

### The Present Study

In the present behavioral study, we adapted the study of Fabre et al. (2015) and focused our analyses on the effect of the gender stereotypical beliefs on the proposers’ economic strategic behavior. Participants played a repeated one-shot ultimatum game as proposers against 120 simulated different responders. The latter were introduced to the participants by occupational nouns stereotypically marked with gender paired with either feminine or masculine proper names (e.g., *linguist-Anna; economist-David; economist-Cristina; linguist-Leonardo*; Fabre et al., 2015). We assumed that reading occupational role nouns stereotypically marked with gender leads to automatic and hard-to-suppress activation of gender stereotypical beliefs (Banaji and Hardin, 1996; Irmen and Roßberg, 2004; Oakhill et al., 2005).

We hypothesized that (1) participants would assign a higher minimum acceptable to both male responders and male-stereotyped responders (i.e., described with an occupation stereotypically marked with male gender) – who may be associated with a higher social status – than to respectively female responders and female-stereotyped responders (i.e., described with an occupation stereotypically marked with female gender), who may in turn be associated with a lower social status (Eckel and Ball, 1996; Rudman and Kilianski, 2000; Ridgeway, 2001). We also hypothesized that (2) proposers would be faster in making their offers to both male and male-stereotyped responders following social norms (i.e., equity rule), while they would take more time to decide when interacting with respectively female and female-stereotyped responders following strategic concerns. According to the *Status Incongruity Hypothesis* (Rudman et al., 2012), socially *atypical* male and female individuals (i.e., not conformant to gender rules; Eagly and Karau, 2002), are judged more negatively than socially *typical* ones, all other things being equal, and may sometimes undergo penalties (i.e., *backlash effect*; Rudman and Glick, 1999; Eagly and Karau, 2002; Rudman and Fairchild, 2004; Phelan et al., 2008; Rudman et al., 2012). Hence, we finally hypothesized that (3) proposers would make higher offers to responders who conform to gender rules (e.g., *linguist-Cristina*, e*conomist-Leonardo*), than to responders who violate gender rules (e.g., *linguist-David*, *economist-Anna)*.

## Materials and Methods

### Participants

Thirty-four students of Modena University (17 females; age range 19–26 years *M* = 21.5, *SD* = 2.26) were recruited to play a repeated one-shot ultimatum game as proposer. They participated for 5% of the total amount of money they won and were proposed at the end of the experiment to swap this money for course credits. All were Italian native speakers with normal or corrected-to-normal vision. None of them reported a history of prior neurological disorder. Participants were informed of their rights and gave written informed consent for participation in the study. This study was carried out fulfilling ethical requirements in accordance with the standard procedures of the University of Modena and Reggio Emilia.

### Materials

The same two groups of 30 occupational role nouns each (one male, one female) with comparable stereotypicality, wealth and valence, lexical frequency and length used in the study of Fabre et al. (2015) were used in the present study (see Supplementary Material). In order to select the experimental materials, a written questionnaire listing 258 occupational role nouns, ending in –e,–ista or a consonant to avoid cues to the gender of the referent in the word form, was presented to 112 students not further involved in the experiment (56 females; age range 19–27 years; *Mage* = 23.6, *SD* = 2.92). Eighty of these students rated to what extent each role noun was stereotypically associated with male or female individuals on a 7-point Likert scale (i.e., stereotype strength; from 1 = only men to 7 = only women), 16 of them to what extent each role noun was associated with a positive or negative value (i.e., valence: from 1 = very negative to 7 = very positive) and 16 the wealth of a person described with each role noun (i.e., wealth: from 1 = very rich to 7 = very poor). The labels of the scale poles were reversed for half of the participants. The final rating assigned to each word was calculated by combining the ratings obtained with both directions of each rating scale. The 60 role nouns selected as experimental materials received comparably high ratings of stereotypicality (the experimental material and the associated ratings are available in Supplementary Material). In order to compare the stereotype strength of the two role noun groups, the ratings of the role nouns ranging from 4 to 7 (i.e., feminine stereotypes) were translated and ranged from 1 to 4 (i.e., X′ = 8-X, with X: initial rating and X′: translated rating). Stereotype strength (Female Stereotypes: *M* = 2.81, *SD* = 1.21; Male Stereotypes: *M* = 2.77, *SD* = 1.19), valence (Female Stereotypes: *M* = 4.42, *SD* = 0.69; Male Stereotypes: *M* = 4.36, *SD* = 0.71), wealth (Female Stereotypes: *M* = 3.81, *SD* = 0.94; Male Stereotypes *M* = 4.10, *SD* = 0.88), lexical frequency (Female Stereotypes: *M* = 5.66, *SD* = 0.87; Male Stereotypes *M* = 6.09, *SD* = 1.05) and length (i.e., number of characters; Female Stereotypes: *M* = 9.77, *SD* = 2.2; Male Stereotypes *M* = 8.83, *SD* = 1.82) of male and female occupational role nouns were comparable (*ps* > 0.05). The mean stereotypicality rating of feminine role nouns reported in the Supplementary Material Table S1 is the translated rating (i.e., X′).

Experimental materials also included 120 Italian familiar proper names (60 feminine) without any unisex names. With the final two groups of 30 occupational role nouns along with the 120 proper names, we created four experimental conditions, two stereotype-matching conditions: female stereotypical occupational role nouns followed by feminine proper names (e.g., *linguista*-*Anna*) and male stereotypical occupational role nouns followed by masculine proper names (e.g., *economista-Davide*); and two stereotype-mismatching conditions: female stereotypical occupational role nouns followed by masculine proper names (e.g., *linguista-Leonardo*) and male stereotypical role nouns followed by feminine proper names (e.g., *economista*-*Cristina*). Participants interacted once with 30 different responders of each kind (i.e., 120 different responders in total).

### Procedure

Participants were seated comfortably in a darkened sound-attenuated room. They played a one-shot ultimatum game as proposers. An introduction explaining the rules of the ultimatum game was given to each of them. Stimuli were presented in light white upper case letters (Courier font, size 13) against a black background on a high-resolution computer that was positioned at eye level about 70 cm in front of each participant. A fixation cross appeared in the middle of a computer screen and remained until participants pressed a button to start a trial. Each occupational role noun was displayed for 700 ms followed by a blank screen for 300 ms. Then a proper name appeared and remained on the screen until the participants pressed the key on the keyboard corresponding to the numerical value of the offer they wanted to make (i.e., from 1€ to 9€ out of 10€). Each response was followed by a 1000 ms blank screen. No feedback on the responder’s answer was provided to the participants in order to avoid a modulation of their behavior along the experiment. Participants were asked to respond as fast as possible.

Before conducting the game, participants were told that they were playing against 120 real different responders of whom they would know their occupations and proper names. Each participant was presented with 30 trials in each of the four experimental conditions for a total of 120 trials. As a matter of fact, the responder was simulated by the computer. However, in order to make the participants believe they were playing against real responders, they were told that responders had been contacted prior to the game and that they had indicated the offers they were willing to accept if proposed by a student (i.e., a shifted in time ultimatum game). Moreover, participants were indicated that we were thanking the different partners involved in this experiment (e.g., the firefighters of Modena, the Oenology School of UNIMORE University, etc…). Participants were informed that at the end of the game, responders would receive the sum corresponding to a percentage of the accepted offers.

### Data Analysis

#### Mean Offers

Mean offers were submitted to a 2 × 2 × 2 (Responders’ Occupation [male-stereotyped, female-stereotyped] × Responders’ Gender [male, female] × Participants’ Gender [male, female]) analysis of variance (ANOVA). Participants’ Gender was a between-subject factor, and the two remaining factors were within-subject factors.

#### Response Times

Log transformed mean response times were submitted to a 2 × 2 × 2 (Responders’ Occupation [male-stereotyped, female-stereotyped] × Responders’ Gender [male, female] × Participants’ Gender [male, female]) ANOVA. Participants’ Gender was a between-subject factor, and the two remaining factors were within-subject factors.

#### Questionnaires

The *Interpersonal Reactivity Index* (IRI; Albiero et al., 2006) was designed to measure empathy and is composed of four subscales: (1) the Perspective Taking scale (pt) measuring the tendency to spontaneously adopt the psychological point of view of others; (2) the Empathic Concern scale (ec) assessing “other-oriented” feelings of sympathy and concern for unfortunate others; (3) the Personal Distress scale (pd) measuring “self-oriented” feelings of personal anxiety and unease in tense interpersonal setting; and (4) the Fantasy scale (f) that taps respondents’ tendencies to transpose themselves imaginatively into the feelings and actions of fictitious characters in books, movies, and plays. The *Bem Sex Role Inventory* (BSRI; De Leo and Villa, 1986) assesses the participants’ degree of masculinity/femininity and to what extent they embrace traditional sex roles. We examined whether the scores at the IRI and the BSRI questionnaires predicted the differences in mean offer and response time observed for the different responders. To that aim, we conducted Pearson correlation analyses between the scores of the IRI, the BSRI, and the resultants of the differences in mean offer and response times participants for (1) responders’ occupations marked with male vs. female stereotypes (i.e., Stereotype [M – F]); and (2) male vs. female responders (i.e., Gender [M – F]).

#### *Post hoc* Rating Study of Social Status

In order to further our argumentation, a final rating study was realized aiming at measuring the social status associated with the occupational stereotypes used in the present study. Fifty one participants (24 females; *Mage* = 31.40, *SD* = 6.11) were asked to rate the social status associated with the individuals practicing each of the 60 occupations used in our experiment (i.e., from 1 = very low social status to 7 = very high social status). This study was realized online via Google Forms. In order to evaluate the impact of the social status associated with the occupational stereotypes on the proposers’ decision-making process, we run two one-tailed partial correlations: one between the occupations stereotypicality (from 1 = very masculine to 7 = very feminine) and the mean offer and one between the occupations stereotipicality and the response times, each time controlling for social status. These results were compared to the results same correlations analysis not controlled for social status.

## Results

### Mean Offer

**FIGURE 1:**
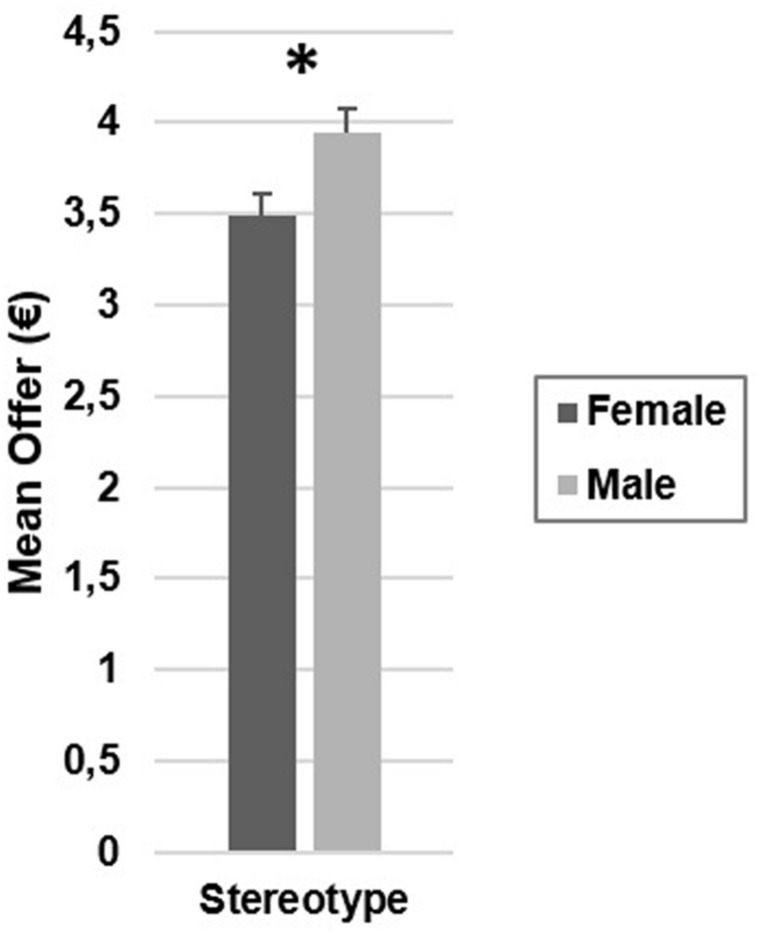
**Mean offers as a function of responders’ gender stereotypes.** Error bars represent standard errors. ^∗^*p* < 0.001.

On average, participants proposed 3.72€ (*SD* = 0.17) to the responders (see **Table 1**). The ANOVA on mean offers showed a main effect of stereotype [*F*(1,32) = 52.53, *p* < 0.001, $\eta_{p}^{2}$ = 0.62, see **Figure 1**]. Participants proposed higher offers to responders presented with male than with female stereotypical occupational role nouns (*M* = 3.94€, *SD* = 0.11; *M* = 3.49€, *SD* = 0.13; respectively). Participants’ gender [*F*(1,32) = 0.30, *p* = 0.59, $\eta_{p}^{2}$ = 0.01], responders’ gender [*F*(1,32) = 0.05, *p* = 0.82, $\eta_{p}^{2}$ = 0.00] main effects and Participants’ Gender × Responders’ Gender [*F*(1,32) = 1.60, *p* = 0.22, $\eta_{p}^{2}$ = 0.05], Stereotype × Participants’ Gender [*F*(1,32) = 0.04, *p* = 0.84, $\eta_{p}^{2}$ = 0.00], Stereotype × Responders’ Gender [*F*(1,32) = 0.06, *p* = 0.82, $\eta_{p}^{2}$ = 0.00], Stereotype × Responders’ Gender × Participants’ Gender [*F*(1,32) = 1.76, *p* = 0.19, $\eta_{p}^{2}$ = 0.05] interactions were not significant.

**Table 1:** Offers and response times means and standard deviations for each experimental condition.

		Female stereotype				Male stereotype			
		Female gender		Male gender		Female gender		Male gender	
		*M*	*SD*	*M*	*SD*	*M*	*SD*	*M*	*SD*
Mean offer (€)									
Female Participants		3.40	*0.72*	3.47	*0.69*	3.87	*0.86*	3.87	*0.86*
Male participants		3.58	*0.59*	3.53	*0.55*	4.01	*0.66*	4.01	*0.59*
Response times (ms)									
Female participants		1359	*977*	1081	*770*	947	*657*	792	*464*
Male participants		1193	*494*	936	*273*	884	*339*	764	*343*

*Italic values are de standard deviations associated with the mean offers and the mean response times.*

### Response Times

**FIGURE 2:**
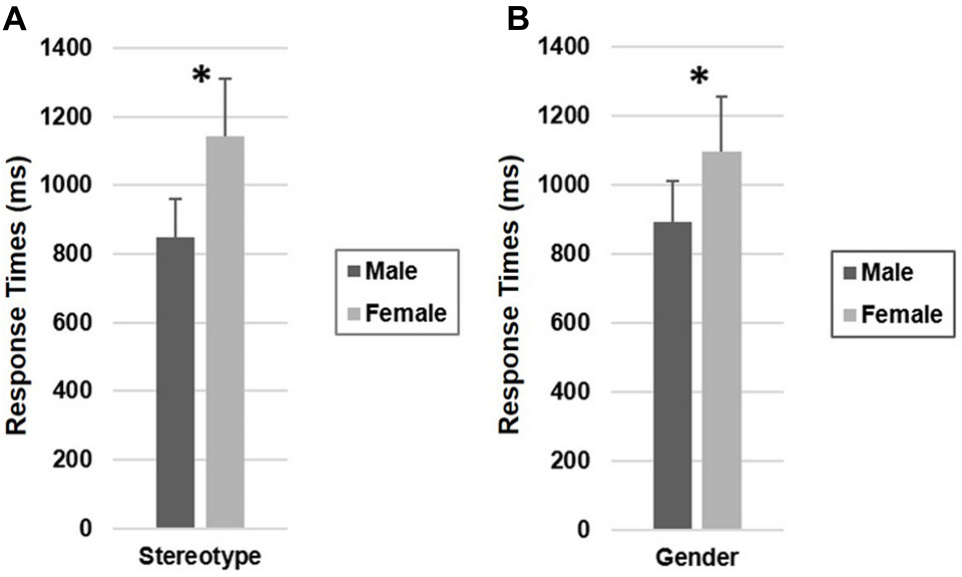
**(A,B)** Response times as a function of **(A)** responders’ gender stereotypes and of **(B)** responders’ genders. Error bars represent standard errors. ^∗^*p* < 0.001.

The ANOVA on log-transformed response times showed a significant stereotype main effect [*F*(1,32) = 43.87, *p* < 0.001, $\eta_{p}^{2}$ = 0.58, see **Figure 2A**] with participants making their offers faster to male-stereotyped responders than to female-stereotyped responders (respectively, *M* = 847 ms, *SD* = 477; *M* = 1142 ms, *SD* = 706, see **Table 1**). Participants were also faster when making their offers to male responders than to female responders (respectively, *M* = 893 ms, *SD* = 520; *M* = 1096 ms, *SD* = 693), as shown by a significant responder’s gender main effect [*F*(1,32) = 54.95, *p* < 0.001, $\eta_{p}^{2}$ = 0.63, see **Figure 2B**]. The participants’ gender [*F*(1,32) = 0.00, *p* = 0.96, $\eta_{p}^{2}$ = 0.00] main effect and Participants’ Gender × Responders’ Gender [*F*(1,32) = 0.04, *p* = 0.85, $\eta_{p}^{2}$ = 0.00], Stereotype × Participants’ Gender [*F*(1,32) = 0.02, *p* = 0.86, $\eta_{p}^{2}$ = 0.00], Stereotype × Responders’ Gender [*F*(1,32) = 2.66, *p* = 0.11, $\eta_{p}^{2}$ = 0.08], Stereotype × Responders’ Gender × Participants’ Gender [*F*(1,32) = 0.25, *p* = 0.62, $\eta_{p}^{2}$ = 0.01] interactions were not significant.

### Questionnaires

The correlations between the scores obtained by each participant in the BSRI and in the IRI and the resultants of the various differences in mean offer and in response time revealed only one significant result (see Supplementary Material). The difference in response times between responders characterized by a male vs. female stereotype [Stereotype (M – F)] was positively correlated to the score of the perceptive taking scale: the higher the score, the greater the Stereotype (M – F) difference [*r* = 0.393, *p* < 0.05, see **Table 2**].

**Table 2:** Correlations between the questionnaires’ scores and the differences in mean offers and response times.

	Questionnaires					Mean offer		Response Times	
	Interpersonal Reactivity Index				Bem sex	Stereotype (M – F)	Gender (M – F)	Stereotype (M – F)	Gender (M – F)
					Role Inventory				
	PT	EC	DP	F					
**Questionnaires**									
IRI (pt)	_____								
IRI (ec)	0.318	_____							
IRI (dp)	**-0.456^∗∗^**	0.103	_____						
IRI (f)	0.175	**0.517^∗∗^**	105	_____					
BSRI	-0.140	-0.226	-0.314	-0.429^∗^	_____				
**Mean offer**									
Stereotype (M – F)	0.090	-0.092	-0.090	0.118	0.056	_____			
Gender (M – F)	-0.033	0.061	0.192	-0.139	-0.135	-0.184	_____		
**Response times**									
Stereotype (M – F)	0.393^∗^	0.198	-0.090	0.000	-0.042	-0.174	-0.020	_____	
Gender (M – F)	-0.205	0.033	0.227	-0.096	0.058	-0.194	-0.008	**0.340^∗^**	_____

*Bold values are statistically significant.*

### *Post hoc* Rating Study of Social Status

A dependent *t*-test analysis was conducted on the social status ratings revealing that the thirty occupations stereotypically male were on average associated with a higher social status (*M* = 4.19, *SD* = 0.60) than are the 30 occupations stereotypically female [*M* = 3.60, *SD* = 0.61, *t*(50) = 10.41, *p* < 0.001]. The occupations stereotypicality and the mean offer were significantly correlated (*r* = -0.396, *p* < 0.001), however, the significance dropped when controlling for social status (*r* = -0.225, *p* < 0.05). The occupational stereotypicality and the response times were also found to be significantly correlated (*r* = 0.507, *p* < 0.001) and lightly less when controlling for social status (*r* = 0.492, *p* < 0.001, see Supplementary Material).

## Discussion

In the present study we investigated whether both the responders’ stereotypical identity and gender modulated the behavior of proposers playing a repeated one-shot ultimatum game. We predicted to observe a modulation of both mean offers and response times depending on the social description of the responders (i.e., gender and occupational stereotype marked with gender).

On average, participants proposed 37.2% of the total amount of money to the responders, which is slightly less than the average offer, i.e., 40% of the share, reported in the meta-analysis of Oosterbeek et al. (2004). This difference is explained by the fact that while participants offered male-stereotyped responders a share similar to the one usually proposed in the ultimatum game (i.e., about 4€ out of 10€; Oosterbeek et al., 2004), they offered female-stereotyped responders on average 45 cents less. The results also revealed that the mean offer was correlated with the degree of stereotypicality of the responders’ occupations. Indeed, the more masculine the occupation was, the higher was the offer; and the more feminine the occupation was, the lower was the offer. These results support the idea that female-stereotyped responders are associated with a lower minimum acceptable offer than are male-stereotyped responders. This difference in behavior appears to be partly due to the fact that male-stereotyped occupations are on average associated with a higher social status compared to male-stereotyped occupations according to both our *post hoc* rating study and literature (Eagly, 1987; Ridgeway, 2001). Participants were also longer to make their offers to female-stereotyped responders than to male-stereotyped responders. Again, the correlation analysis supported the results of the analysis of variance and showed that response times were correlated with the stereotypicality of the responders’ occupations. The more masculine the occupation was, the shorter were the response times; and the more feminine the occupation was, the longer were the response times. Taken together, these results suggest that participants behaved more or less strategically depending on the responders’ stereotypical identity. When facing male-stereotyped responders (e.g., economist), proposers followed social norms, applying the equity rule, offering quickly fair shares. In contrast, when facing female-stereotyped responders, proposers adopted a more strategic and cognitively costly deliberative reasoning, trying to accurately determine their minimum acceptable offer in order to maximize their gain. These behaviors were more extreme when the gender stereotype strength was higher: the more feminine the occupational stereotype was, the more strategic was the decision; and the more masculine the occupational stereotype was, the more altruistic was the decision.

Participants were also found to take more time to make an offer to female responders than to male responders, to this extent it may have been more complex for participants to interact with female responders than with male responders. However, in contradiction with our predictions, participants made comparable offers to both female and male responders. We assume that offering female responders shares equivalent to those offered to male responders, may have had a cognitive cost for participants. The increase in response times may reflect a strategic behavior inhibition when interacting with female responders.

Finally, our predictions concerning the observation of a backlash effect directed at the responders who violate gender rules (e.g., economist-Anna; linguist-David) were not fulfilled, since the results revealed no economical penalization toward these specific responders. The studies reporting backlash effects used diverse experimental protocols testing either the fit of hiring (e.g., Rudman and Glick, 2001; Heilman et al., 2004; Heilman and Okimoto, 2007; Moss-Racusin et al., 2010; Rudman et al., 2012), the selection of a partner game (e.g., Rudman, 1998), the salary recommendation (e.g., Heilman et al., 2004) or the opportunity to sabotage a line manager (e.g., Rudman et al., 2012), to name a few (for a review on backlash effect see Rudman and Phelan, 2008). In these studies, participants were given the possibility to commit backlash but were not taking any risk in doing so. Indeed, the backlashed individuals were not able to punish the participants in return for their behavior. We assume that no backlash effect was observed in the present experiment because participants may have feared to be punished by the responders, who may have rejected their offer for being backlashed in the first place. A second possibility might be that participants were simply not willing to backlash mismatching responders. The present study does not enable to status on the absence of backlash effect. We plan to address this question in further studies.

## Conclusion

The present study continues the long list of works investigating the impact of social information on economic decision-making. As far as we know, this study is the first to demonstrate that both men and women modulate their strategic behavior according the gender-marked stereotype of their counterparts during economic interactions. Proposers were found to apply quickly the equity rule when interacting with male-stereotyped responders, while they behaved more strategically at a greater cognitive cost with female-stereotyped responders proposing them lower shares. Proposers were longer to make their offers to female than to male responders but both kinds of responders received comparable offers, suggesting a greater cognitive effort to treat females as equally as males. Taken together, these results suggest that in real life, individuals practicing a profession stereotypically female may suffer discrimination during economic interactions, while female individuals may not, at least when these individuals are given the possibility to punish in return their counterpart. The present experiment highlights an evolution of society in that gender discrimination, which is nowadays strongly decried, may be converting into a more insidious discrimination toward individuals with female characteristics. More work is now needed to confirm this tendency.

## Conflict of Interest Statement

The authors declare that the research was conducted in the absence of any commercial or financial relationships that could be construed as a potential conflict of interest.
