# Supplementary material for: The Responders’ Gender Stereotypes Modulate the Strategic Decision-Making of Proposers Playing the Ultimatum Game
Source: Front Psychol. 2016 Jan 25;7:12. doi: 10.3389/fpsyg.2016.00012 (PMC4724784; doi:10.3389/fpsyg.2016.00012)
Supplement: Supplementary file 1 [file Supplementary_1.DOCX]

***Supplementary 1.*** *Stereotypical strength, valence and wealth ratings associated with the sixty occupational role nouns stereotypically marked with gender selected for this experiment (standard deviation)*
