# Supplementary figures and images for: The Responders’ Gender Stereotypes Modulate the Strategic Decision-Making of Proposers Playing the Ultimatum Game
Source: Front Psychol. 2016 Jan 25;7:12. doi: 10.3389/fpsyg.2016.00012 (PMC4724784; doi:10.3389/fpsyg.2016.00012)

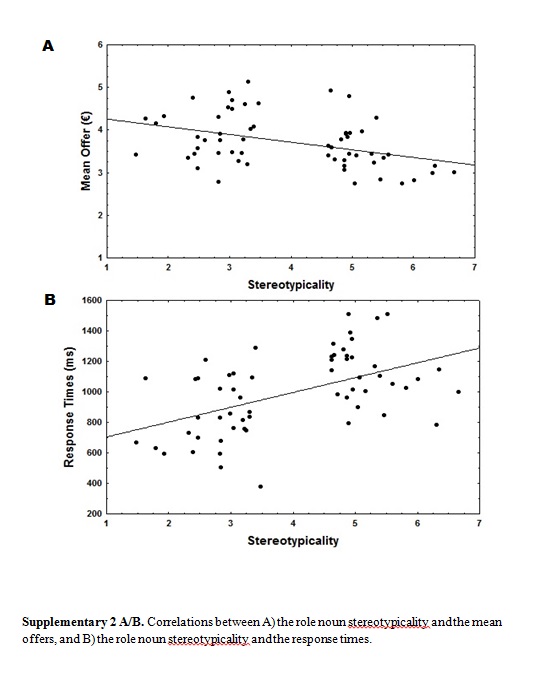

Supplement: Supplementary file 2 [file Supplementary_2.JPEG]
